# Genotypic and phenotypic landscape of carbapenem-resistant *Pseudomonas aeruginosa* isolated from respiratory and non-respiratory samples in a tertiary hospital

**DOI:** 10.1186/s12866-026-04756-8

**Published:** 2026-03-14

**Authors:** Hamiyet Büşra Gündoğdu, Erva Rakıcı, Nebahat Ejder, Ayşegül Çopur Çiçek, Osman Birol Özgümüş

**Affiliations:** 1 Erzurum Public Health Laboratory, Erzurum, Turkey; 2 Department of Medical Microbiology, Faculty of Medicine, Recep Tayyip Erdogan University, Rize, Turkey; 3 Department of Medical Microbiology, Istanbul Medipol University Faculty of Medicine, Istanbul, Turkey

**Keywords:** Pseudomonas aeruginosa, CRPA, Carbapenem resistance, ExoT, ExoY, PFGE, Fisher’s exact test, FDR, BH, EUCAST, Reproducible research

## Abstract

**Background:**

The World Health Organization lists carbapenem-resistant *Pseudomonas aeruginosa* (CRPA) as a critical priority pathogen. However, the links between resistance phenotypes, virulence factors, and clonal spread remain incompletely understood. We aimed to characterize the genotypic and phenotypic landscape of clinical CRPA isolates and evaluate whether specimen source or type III secretion effectors (*exoT*/*exoY*) serve as predictors of antibiotic resistance.

**Methods:**

Fifty-eight consecutive CRPA isolates from respiratory and non-respiratory specimens were analyzed. Susceptibility to 10 antimicrobial agents was determined using an automated system. PCR was used to screen for seven carbapenemase genes, six virulence/quorum-sensing genes, and the efflux marker *mexY*. Macrorestriction patterns were typed by *Spe*I-PFGE. Statistical associations were assessed using two-tailed Fisher’s exact tests with Benjamini–Hochberg false-discovery-rate (FDR) correction (α = 0.05).

**Results:**

Resistance rates were highest for piperacillin/tazobactam (91%), ceftazidime (81%), and cefepime (81%); notably, 34% of isolates exhibited a pan-drug-resistant (PDR) profile. Only three isolates (5%) carried *bla*VIM; no other carbapenemase genes were detected. Virulence markers were highly prevalent (*exoY* 66%, *exoT* 57%, *algD* 45%; *lasR* 91%, *rhlR* 95%). After FDR adjustment, neither virulence gene presence (*exoT*, *exoY*) nor specimen origin correlated significantly with resistance to β-lactams, aminoglycosides, or fluoroquinolones (lowest *q* = 0.093). Furthermore, gene prevalence did not differ significantly between respiratory and non-respiratory isolates. PFGE analysis revealed 41 distinct pulsotypes without a dominant clone, suggesting sporadic horizontal gene transfer rather than clonal expansion.

**Conclusions:**

This CRPA cohort is genetically diverse, multidrug-resistant, and lacks anatomical segregation by genotype. The presence of *exoT*/*exoY* does not appear to shape resistance phenotypes in this setting. Infection control strategies should prioritize the containment of mobile genetic elements and implement genome-based surveillance, rather than focusing solely on specific clones or infection sites.

**Supplementary Information:**

The online version contains supplementary material available at 10.1186/s12866-026-04756-8.

## Introduction

*Pseudomonas aeruginosa* is a leading Gram-negative pathogen responsible for healthcare-associated infections, particularly in hospitalized patients. The escalating resistance of this pathogen complicates the treatment of infections in the respiratory and urinary tracts, skin, and soft tissues, leading to increased morbidity, mortality, and healthcare costs [1].

While Carbapenem-Resistant *P. aeruginosa* (CRPA) can be isolated from diverse anatomical sites, respiratory isolates are frequently implicated in severe conditions such as ventilator-associated pneumonia (VAP) and hospital-acquired pneumonia (HAP) [2]. These strains are prevalent among mechanically ventilated patients in intensive care units (ICUs) and often exhibit broader antibiotic resistance profiles, facilitating their persistence in the hospital environment [3].

The pathogenicity of *P. aeruginosa* is driven by a multifactorial arsenal, including antibiotic resistance mechanisms, immune evasion strategies, biofilm formation, and cytotoxicity [4]. In recent years, the rise of multidrug-resistant (MDR) strains has severely limited therapeutic options [5]. Carbapenem resistance in *P. aeruginosa* is primarily driven by chromosomal mechanisms, such as intrinsic β-lactamase overexpression, outer membrane porin loss (e.g., OprD), and efflux pump upregulation [6]. Furthermore, the acquisition of plasmid-mediated carbapenemase genes facilitates the rapid global dissemination of resistance [7]. Given this alarming trend, rigorous surveillance of resistance and virulence determinants is essential for successful infection management [8].

Carbapenemase production is of particular concern as it enables rapid spread via mobile genetic elements [9]. *P. aeruginosa* can harbor class A, B, and D carbapenemases, with class B metallo-β-lactamases (MBLs) such as VIM (Verona integron-encoded metallo-β-lactamase), IMP (Imipenemase), and NDM (New Delhi metallo-β-lactamase) being the most common [10]. Additionally, multidrug efflux pumps of the Resistance-Nodulation-Cell Division (RND) superfamily play a pivotal role in both intrinsic and acquired resistance. Notably, the MexXY pump mediates resistance to aminoglycosides, fluoroquinolones, and cefepime [11].

Virulence in *P. aeruginosa* is often regulated by Quorum Sensing (QS) systems, including *las* and *rhl*, which coordinate the expression of factors such as biofilm components (*algD*) and Type III Secretion System (T3SS) effectors like *exoT* and *exoY* [12]. Understanding the molecular epidemiology of these resistance and virulence genes is critical for characterizing genetic diversity and designing effective infection control strategies [13].

The aim of this study was to evaluate the antibiotic susceptibility profiles of respiratory and non-respiratory clinical CRPA isolates, screen for key resistance and virulence genes, investigate potential correlations between genotype and phenotype, and analyze the clonal relatedness of the isolates.

## Materials and methods

### Study isolates

Between July 2018 and March 2021, we collected 58 non-duplicate, carbapenem-resistant Pseudomonas aeruginosa (CRPA) isolates, defined as resistant to imipenem and/or meropenem according to EUCAST 2021 break-points [14]. Each isolate represented a single patient in one of three clinical settings: intensive-care units (ICU, 29/58 = 50%), general hospital wards (19/58 = 33%), or out-patient clinics (10/58 = 17%). Specimen sources comprised respiratory tract samples—sputum, tracheal aspirate, or broncho-alveolar lavage—(22/58 = 38%) and non-respiratory samples (36/58 = 62%), the latter including tissue or wound swabs (12/58 = 21%), urine (10/58 = 17%), ear discharge (7/58 = 12%), blood or catheter tips (5/58 = 9%), and sterile body fluid obtained by paracentesis (2/58 = 3%). Species identification and preliminary antimicrobial-susceptibility testing were performed with the VITEK-2 Compact system (bioMérieux, France). Isolate-level epidemiological and molecular characteristics (location, specimen type, phenotypic resistance, virulence and quorum-sensing markers, and PFGE phylotypes) are summarized in Table 1.

**Table 1:** Epidemiological and molecular characteristics of the 58 CRPA isolates analysed in this study. Rows are ordered first by specimen group (top panel, respiratory; bottom panel, non-respiratory) and then by isolate code (H1–H58). For each isolate we report the hospital location, specimen source, phenotypic resistance pattern

Pseudomonas aeruginosa isolate		Hospital Unit	Source	Antibiotic Resistance Pattern	Resistance Gene(s)	Virulence Gene(s)	Quorum Sensing Gene(s)	PFGE Phylotype
Respiratory tract sample (*n* = 22)	H4	Anaesthesia ICU	TA	IPM, MEM, CAZ, FEP, TZP	*mexY*	*-*	*lasR*,* rhlR*	ND
	H5	Surgical ICU	Sputum	PDR	*mexY*	*exoT*	*lasR*,* rhlR*	1
	H7	Surgical ICU	TA	IPM, MEM, CAZ, FEP, CIP, LEV, TZP	*mexY*	*-*	*lasR*,* rhlR*	23
	H12	Surgical ICU	TA	IPM, MEM, CIP, LEV, TZP	*mexY*	*exoT*,* exoY*	*lasR*,* rhlR*	14
	H14	Chest diseases service	Lavage	PDR	*mexY*	*exoT*	*lasR*,* rhlR*	3b
	H17	Surgical ICU	TA	PDR	*-*	*rhlR*	*rhlR*	32
	H24	Surgical ICU	TA	PDR	*-*	*exoY*	*lasR*	31a
	H26	Internal medicine ICU	TA	IPM, MEM, CAZ, FEP, TZP	*-*	*exoT*,* exoY*	*lasR*,* rhlR*	36
	H27	Surgical ICU	TA	IPM, MEM, CAZ, FEP, CIP, LEV, TZP	*-*	*exoT*,* exoY*	*lasR*,* rhlR*	40
	H28	Internal medicine ICU	TA	PDR	*bla*_*VIM*_	*exoT*,* exoY*	*lasR*,* rhlR*	22
	H31	Internal medicine ICU	TA	PDR	*-*	*exoT*,* algD*,* exoY*	*lasR*,* rhlR*	2
	H34	Chest diseases service	TA	IPM, MEM, TZP	*-*	*exoT*,* algD*,* exoY*	*lasR*,* rhlR*	27
	H36	Surgical ICU	TA	PDR	*-*	*exoY*	*rhlR*	ND
	H41	Internal medicine ICU	TA	IPM, MEM, CN, TOB, CAZ, FEP, CIP, LEV, TZP	*bla*_*VIM*_, *mexY*	*exoT*,* exoY*,* exoA*	*lasR*,* rhlR*	12
	H42	2nd line ICU	Sputum	IPM, MEM, CAZ, FEP, CIP, LEV, TZP	*mexY*	*exoT*,* algD*,* exoY*	*lasR*,* rhlR*	16
	H43	Internal medicine ICU	TA	IPM, MEM, CAZ, FEP, CIP, LEV, TZP	*-*	*exoT*,* algD*,* exoY*	*lasR*,* rhlR*	17
	H44	Chest diseases service	TA	IPM, MEM, TZP	*mexY*	*exoT*,* exoY*	*lasR*,* rhlR*	37
	H45	Internal medicine ICU	TA	IPM, MEM, CAZ, FEP, LEV, TZP	*mexY*	*algD*,* exoY*	*lasR*,* rhlR*	19
	H47	Chest diseases service	Sputum	IPM, CAZ, FEP, TZP	*mexY*	*exoT*,* algD*,* exoY*	*lasR*,* rhlR*	24
	H48	Anaesthesia ICU	TA	PDR	*bla*_*VIM*_	*exoT*,* algD*,* exoY*,* exoA*	*lasR*,* rhlR*	13
	H50	Surgical ICU	TA	IPM, MEM	*-*	*algD*,* exoY*,* exoA*	*lasR*,* rhlR*	25
	H55	Covid service-9 (brain surgery)	TA	IPM, MEM, CN, CAZ, FEP, CIP, LEV, TZP	*-*	*exoT*,* algD*,* exoY*,* exoA*	*lasR*,* rhlR*	38b
Non-Respiratory tract sample (*n* = 36)	H1	Orthopaedic service	Tissue	IPM, MEM, CN, CAZ, FEP, CIP, LEV, TZP	*mexY*	*exoT*	*lasR*,* rhlR*	8
	H2	Anaesthesia ICU	Blood/Catheter	PDR	*-*	*algD*	*lasR*,* rhlR*	4
	H3	Internal medicine service	Wound swab	PDR	*mexY*	*-*	*lasR*,* rhlR*	1
	H6	Internal medicine ICU	Blood	PDR	*mexY*	*-*	*lasR*,* rhlR*	5
	H8	ENT Outpatient polyclinic	Ear discharge	IPM, MEM, AK, CN, TOB, CAZ, CIP, LEV, TZP	*mexY*	*-*	*lasR*,* rhlR*	23b
	H9	Anaesthesia ICU	Urine	PDR	*mexY*	*-*	*lasR*,* rhlR*	3
	H10	ENT outpatient polyclinic	Ear discharge	PDR	*mexY*	*exoT*	*lasR*,* rhlR*	18
	H11	Plastic surgery polyclinic	Tissue	PDR	*mexY*	*exoT*	*lasR*,* rhlR*	6
	H13	Surgical ICU	Wound swab	PDR	*mexY*	*exoT*,* algD*	*lasR*,* rhlR*	6b
	H15	ENT outpatient polyclinic	Ear discharge	IPM, MEM, AK, CN, TOB, FEP, CIP, LEV, TZP	*mexY*	*algD*	*lasR*,* rhlR*	30
	H16	Internal medicine service	Urine	IPM, MEM, CAZ, FEP, CIP, LEV, TZP	*mexY*	*exoT*,* algD*	*-*	30
	H18	Internal medicine ICU	Tissue	IPM, MEM, CN	*-*	*-*	*-*	34
	H19	Internal medicine ICU	Wound swab	IPM, MEM, CN,	*-*	*exoY*	*-*	30
	H20	Home health services	Urine	IPM, MEM, CAZ, FEP, CIP, LEV, TZP	*-*	*exoT*,* exoY*	*lasR*,* rhlR*	ND
	H21	Internal medicine ICU	Blood	IPM, MEM, CN, CAZ, FEP, LEV, TZP	*-*	*lasR*,* rhlR*	*lasR*,* rhlR*	34
	H22	Internal medicine ICU	Tissue	IPM, MEM, CN, CAZ, FEP, LEV, TZP	*-*	*lasR*,* rhlR*	*lasR*,* rhlR*	34b
	H23	Surgical ICU	Blood	PDR	*-*	*exoY*,* lasR*,* rhlR*	*lasR*,* rhlR*	31
	H25	ENT outpatient polyclinic	Ear discharge	PDR	*-*	*exoT*,* algD*,* exoY*	*lasR*,* rhlR*	33
	H29	Orthopaedic service	Tissue	IPM, MEM, CN, CAZ, FEP, TZP	*-*	*exoT*,* exoY*,* exoA*	*rhlR*	20
	H30	Infectious diseases service	Tissue	PDR	*mexY*	*exoT*,* algD*,* exoY*	*lasR*,* rhlR*	2
	H32	Internal medicine Service	Urine	IPM, MEM, TOB, CAZ, FEP, TZP	*mexY*	*exoT*,* exoY*	*lasR*,* rhlR*	41
	H33	Palliative care unit	Ascites	IPM, MEM	*mexY*	*exoY*	*lasR*,* rhlR*	41a
	H35	Internal medicine service	Wound swab	IPM, MEM	*-*	*algD*,* exoY*	*lasR*,* rhlR*	39
	H37	Internal medicine ICU	Urine	IPM, MEM, CN, TOB, CAZ, FEP, CIP, LEV, TZP	*-*	*exoT*,* exoY*	*lasR*,* rhlR*	35
	H38	Internal medicine polyclinic	Urine	IPM, MEM, CAZ, FEP, LEV, TZP	*-*	*algD*,* exoY*	*lasR*,* rhlR*	9
	H39	Internal medicine service	Urine	IPM, MEM, CAZ, CIP, LEV, TZP	*-*	*exoY*	*lasR*,* rhlR*	10
	H40	ENT outpatient polyclinic	Ear discharge	IPM, MEM, AK, CN, TOB, FEP, CIP, LEV, TZP	*mexY*	*exoT*,* algD*,* exoY*	*lasR*,* rhlR*	15
	H46	ENT outpatient polyclinic	Ear discharge	IPM, MEM, AK, CN, TOB, FEP, CIP, LEV, TZP	*mexY*	*exoT*,* algD*,* exoY*	*lasR*,* rhlR*	21
	H49	Surgical ICU	Tissue	IPM, MEM, CN, TOB, CAZ, FEP, CIP, LEV, TZP	*-*	*algD*,* exoY*,* exoA*	*lasR*,* rhlR*	8b
	H51	Internal medicine service	Catheter	IPM, MEM, CN, TOB, CAZ, FEP, CIP, LEV, TZP	*-*	*exoT*,* algD*	*lasR*,* rhlR*	26
	H52	Internal medicine service	Paracentesis	PDR	*mexY*	*exoT*,* algD*,* exoY*	*lasR*,* rhlR*	26a
	H53	Covid service-9 (brain surgery)	Wound swab	IPM, MEM, CN, CAZ, FEP, CIP, LEV, TZP	*mexY*	*exoT*,* exoY*,* exoA*	*lasR*,* rhlR*	38
	H54	Anaesthesia ICU	Urine	IPM, MEM, CN, CAZ, FEP, TZP	*-*	*exoT*,* algD*,* exoY*	*lasR*,* rhlR*	11
	H56	ENT outpatient polyclinic	Ear discharge	PDR	*mexY*	*algD*,* exoY*,* exoA*	*lasR*,* rhlR*	7
	H57	Chest diseases service	Urine	IPM, MEM, CN, CAZ, FEP, TZP	*-*	*exoT*,* algD*,* exoY*	*lasR*,* rhlR*	28
	H58	Emergency polyclinic	Urine	IPM, MEM, CN, CAZ, LEV, TZP	*-*	*algD*,* exoY*,* exoA*	*lasR*,* rhlR*	29

*IMI *imipenem, *MEM *meropenem, *AK *amikacin, *CN *gentamicin, *TOB *tobramycin, *CAZ *ceftazidime, *FEP *cefepime, *CIP *ciprofloxacin, *LEV *levofloxacin, *TZP *piperacillin/tazobactam, *PDR *pan-drug resistance, the carbapenemase and efflux genes detected (e.g. *bla*_VIM_, *mexY*), virulence genes (e.g. *exoT*, *algD*), quorum-sensing regulators (*lasR*, *rhlR*), and the corresponding PFGE phylotype (PT 1–41; ND, band pattern not obtained for isolates H4, H20, and H36)

### Antimicrobial susceptibility testing

Antimicrobial susceptibility was determined with the VITEK-2 Compact automated system (bioMérieux, France) using the AST-GN13 card (*Pseudomonas* panel, REF 413400). The antibiotic panel comprised imipenem, meropenem, amikacin, gentamicin, tobramycin, ceftazidime, cefepime, ciprofloxacin, levofloxacin and piperacillin/tazobactam. Results were interpreted according to EUCAST breakpoint tables, version 11.0 (2021) (EUCAST 2021) [14].

### Polymerase chain reaction (PCR) assays

Isolates stored in 20% glycerol at − 80 °C were revived on 5% sheep-blood agar. Genomic DNA of the isolates was extracted by the boiling method [15]. six carbapenemase genes—*bla*_IMP_, *bla*_VIM_, *bla*_OXA-48_, *bla*_KPC_, *bla*_NDM_, *bla*_SIM_—; the efflux-associated *mexY*— as well as six virulence or quorum-sensing genes (*exoA*, *exoT*, *exoY*, *algD*, *lasR* and *rhlR*) were detected by in-house conventional PCR. Primer sequences are listed in Table 2; cycling parameters and reaction mixtures are provided in the publications [16–24] in the Supplementary Table S1. Amplicons were resolved on 1% agarose gels (100 V, 60 min) and visualised with a MiniLumi gel documentation system (DNR Bio-Imaging Systems, Israel).

**Table 2:** PCR primer sequences, expected amplicon sizes, and references used in this study

Group Primer	Target Gene	Primer Sequence (5´→ 3´)	Amplicon size (bp)	References
Carbapenemase genes	*bla*_IMP_	F: CATGGTTTGGTGGTTCTTGT R: ATAATTTGGCGGACTTTGGC	488	[16]
	*bla*_VIM_	F: ATTGGTCTATTTGACCGCGTC R: TGCTACTCAACGACTGAGCG	780	[16]
	*bla*_OXA−48_	F: GCGTGGTTAAGGATGAACAC R: CATCAAGTTCAACCCAACCG	442	[17]
	*bla*_KPC_	F: ATGTCACTGTATCGCCGTCT R: TTTTCAGAGCCTTACTGCCC	893	[18]
	*bla*_NDM_	F: GAGATTGCCGAGCGACTTG R: CGAATGTCTGGCAGCACACTT	497	[19]
	*bla*_SIM_	F: TACAAGGGATTCGGCATCG R: TAATGGCCTGTTCCCATGTG	570	[20]
Virulence Factor Genes	*exoA*	F: AACCAGCTCAGCCACATGTC R: CGCTGGCCCATTCGCTCCAGCGCT	396	[21]
	*exoT*	F: AATCGCCGTCCAACTGCATGCG R: TGTTCGCCGAGGTACTGCTC	152	[21]
	*exoY*	F: CGGATTCTATGGCAGGGAGG R: GCCCTTGATGCACTCGACCA	289	[21]
	*algD*	F: ATGCGAATCAGCATCTTTGGT R: CTACCAGCAGATGCCCTCGGC	1311	[22]
Quorum Sensing Genes	*lasR*	F: AAGTGGAAAATTGGAGTGGAG R: GTAGTTGCCGACGACGATGAAG	130	[23]
	*rhlR*	F: TGCATTTTATCGATCAGGGC R: CACTTCCTTTTCCAGGACG	133	[23]
Efflux/Resistance marker	*mexY*	F: CGCCGCAACTGACCCGCTACA R: GGACCACGCCGAAACCGAACG	236	[24]

### Pulsed field gel electrophoresis (PFGE)

The PFGE protocol as previously described [25] was modified and then applied. The DNA extracted from the isolates were digested with *Spe*I enzyme and run for 18 h at 200 V (6 V/cm), 14 °C temperature, 5 s start and 20 s end pulse time in CHEF-DR II electrophoresis device (Bio-Rad Laboratories, Nazareth, Belgium) [25]. *Pseudomonas aeruginosa* ATCC27853 was used as a marker in the evaluation phase. After electrophoresis, the gel was stained with ethidium bromide (Amresco, USA) for 20 min, visualised with a UV transilluminator (Vilber Lourmat, Germany) and then was evaluated according to the principles as described prevously [26]. The gel was photographed with a gel imaging system (DNR MiniLumi, Israel). PyElph 1.3 software [27] was used for dendogram analysis. The proximity distance of the gel bands was determined as 2%.

### Statistical analysis

All analyses were performed in Python 3.11 (Anaconda Inc., Austin TX, USA); descriptive cross-checks were carried out in SPSS v25 (IBM, Chicago IL). Categorical variables were compared with two-tailed Fisher’s exact test [28]. An initial significance level of *p* < 0.05 was applied, and effect size was expressed as an odds ratio (OR) with 95% confidence intervals (CI) calculated by the Woolf log method. Zero-cell tables were adjusted with the Haldane–Anscombe 0.5 continuity correction. To control for multiple testing, raw *p*-values were adjusted with the Benjamini–Hochberg step-up procedure (false-discovery rate 0.05) [29]. Adjusted probabilities are reported as *q*-values, computed with the statsmodels package [30]. Statistical significance was judged after adjustment; hence a comparison was considered significant when *q* < 0.05, while 0.05 ≤ *q* < 0.10 was interpreted as a borderline trend. Plots were generated with Matplotlib v3.8, and descriptive percentages combine the “susceptible” and “intermediate” EUCAST categories. According to EUCAST definitions [14], the ‘Intermediate’ category is interpreted as ‘Susceptible, Increased exposure’. Therefore, for the purpose of statistical binary analysis (Resistant vs. Non-Resistant), ‘Intermediate’ isolates were pooled with ‘Susceptible’ ones to reflect clinical treatability potentials. All Python code, Conda environment specifications used in these analyses are available in Supplementary Material.

### Use of AI-assisted tools

Parts of manuscript preparation were assisted by an artificial-intelligence tool (ChatGPT; OpenAI; accessed August 2025). The tool was used to: (i) assist with English language editing and formatting; (ii) generate and refactor Python code to reproduce the statistical analyses from our raw count tables (two-tailed Fisher’s exact tests; Benjamini–Hochberg false discovery rate adjustment; figure generation with matplotlib); and (iii) check internal consistency among tables, figures, and file names. All outputs were re-run locally and independently verified against the raw data provided in the Supplementary Materials. No confidential or patient-identifying information was entered. The tool had no role in study design, data collection, or the decision to submit and is not listed as an author.

## Results

### Antimicrobial susceptibility testing

**Fig. 1:**
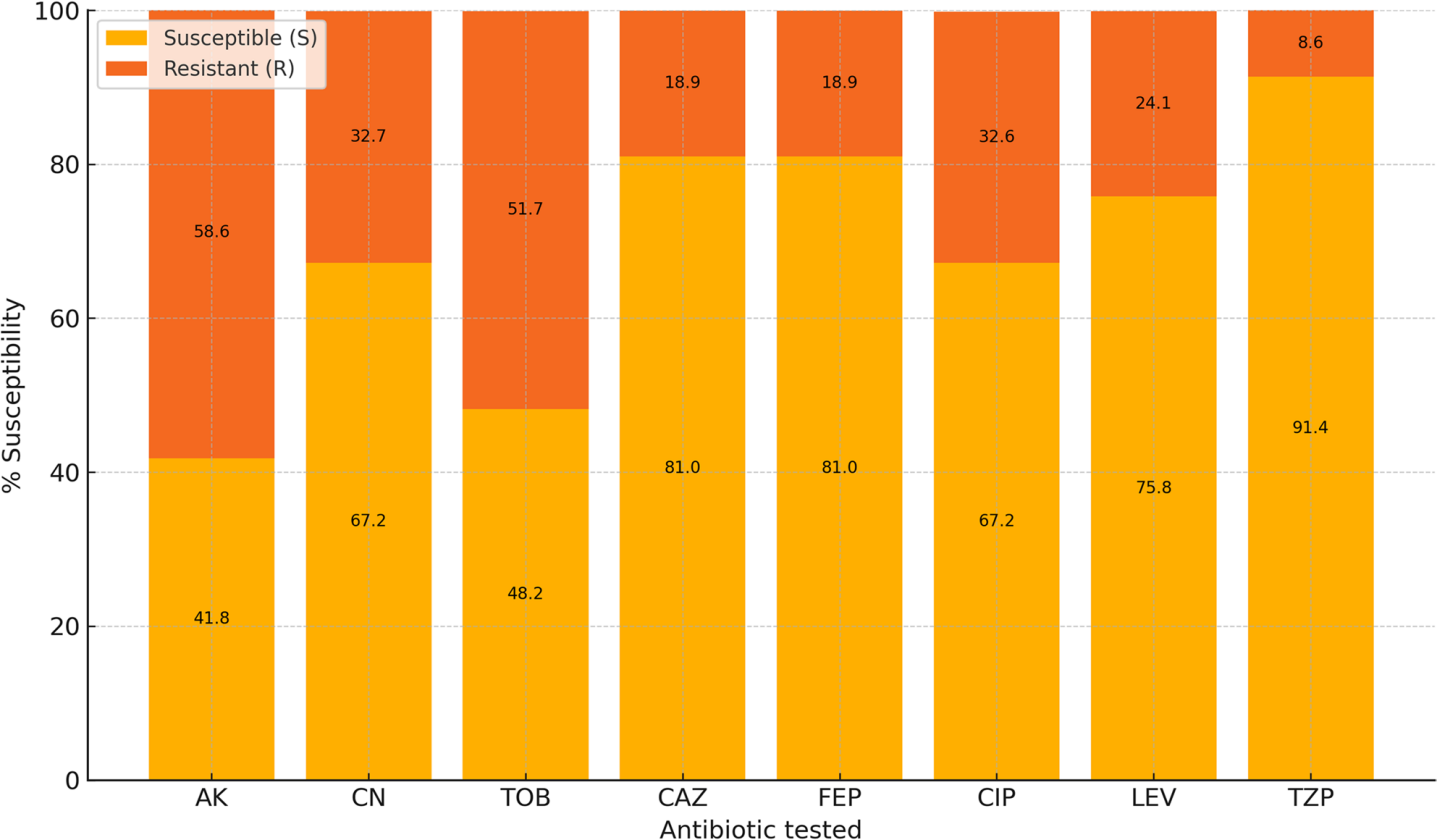
Antimicrobial profile of 58 carbapenem-resistant *Pseudomonas aeruginosa* (CRPA) isolates. Stacked bars show the proportions of resistant (R) and non-resistant isolates (S + I) isolates for each antibiotic. Abbreviations: AK, amikacin; CN, gentamicin; TOB, tobramycin; CAZ, ceftazidime; FEP, cefepime; CIP, ciprofloxacin; LEV, levofloxacin; TZP, piperacillin/tazobactam. Exact percentages are annotated on the bars

Composite susceptibility data are shown in Fig. 1. Isolates with an intermediate were pooled with the susceptible category. Resistance was highest to piperacillin–tazobactam, observed in 53 of 58 isolates (91%). Ceftazidime and cefepime shared the next-highest rates, each resisted by 47 isolates (81%). Twenty isolates (34%) exhibited a pan-drug-resistant profile, i.e. non-susceptibility to all ten agents tested. Detailed isolate-level metadata are shown in Table 1.

### PCR findings

Quorum-sensing regulators *lasR* and *rhlR* were most frequent (53/58; 91% and 55/58; 95%, respectively). The efflux determinant *mexY* was detected in 28/58 isolates (48%).

### Distribution of carbapenemase/virulence genes by sample type

Prevalence of 11 genes in respiratory (*n* = 22) versus non-respiratory (*n* = 36) isolates is summarised in Table 3. Only *bla*_VIM_ appeared more common in respiratory samples in the unadjusted analysis (*p* = 0.046). After Benjamini–Hochberg correction, the lowest *q*-value was 0.65; no comparison remained significant (Supplementary Fig S1). Sample type therefore did not independently explain gene distribution in this cohort.

**Table 3:** Prevalence of resistance- and virulence-associated genes in respiratory versus non-respiratory CRPA isolates

Gene		Respiratory *n*(%)	Non-respiratory *n*(%)	OR	95% CI	*p*	q
*bla*_OXA−48_	0 (0%)	0 (0%)	1.622	0.03–84.68	1.000	1.000	1.000
*bla*_SIM_	0 (0%)	0 (0%)	1.622	0.03–84.68	1.000	1.000	1.000
*bla*_VIM_	3 (14%)	0 (0%)	13.103	0.64–266.83	0.050	0.649	0.649
*bla*_IMP_	0 (0%)	0 (0%)	1.622	0.03–84.68	1.000	1.000	1.000
*bla*_KPC_	0 (0%)	0 (0%)	1.622	0.03–84.68	1.000	1.000	1.000
*bla*_NDM_	0 (0%)	0 (0%)	1.622	0.03–84.68	1.000	1.000	1.000
*exoA*	4 (18%)	5 (14%)	1.393	0.35–5.49	0.718	1.000	1.000
*exoY*	17 (77%)	21 (58%)	2.294	0.72–7.31	0.166	1.000	1.000
*exoT*	15 (68%)	18 (50%)	2.067	0.70–6.11	0.274	1.000	1.000
*algD*	9 (41%)	17 (47%)	0.784	0.27–2.24	0.787	1.000	1.000
*mexY*	10 (45%)	18 (50%)	0.840	0.30–2.38	0.791	1.000	1.000
*lasR*	20 (91%)	33 (92%)	0.857	0.15–4.75	1.000	1.000	1.000
*rhlR*	21 (95%)	34 (94%)	1.039	0.13–8.44	1.000	1.000	1.000

Counts (n) and within-group proportions (%) are shown for each gene among respiratory (*n* = 22) and non-respiratory (*n* = 36) isolates. Odds ratios (OR) and 95% confidence intervals (CI) were calculated with a Haldane–Anscombe 0.5 correction to accommodate zero cells. Two-tailed Fisher’s exact test *p*-values were adjusted for multiple comparisons with the Benjamini–Hochberg procedure (FDR = 0.05); adjusted values are reported as *q*-values. No gene remained significant after FDR correction (lowest *q* = 0.65)

### Relationship between *exoT / exoY* presence and antibiotic susceptibility

Susceptibility to eight antibiotics was compared between *exoT*/*exoY*-positive and -negative subgroups (2 × 2 Fisher’s exact tests, two-tailed; Woolf log OR; BH-FDR across 8 comparisons per gene). For *exoT*, piperacillin/tazobactam showed a nominal association (*p* = 0.012, OR = 17.98, 95% CI 0.94–342.35), but did not remain significant after FDR correction (*q* = 0.093). For *exoY*, gentamicin exhibited a nominal association (*p* = 0.044, OR = 0.24, 95% CI 0.06–0.97), which also did not survive FDR (*q* = 0.351). No *exoT*/*exoY*–antibiotic association remained significant after multiple-testing correction, consistent with Supplementary Fig S2 (BH-adjusted *q*-values) and Table 4 (counts, OR, 95% CI, *p*, *q*).

**Table 4:** Association between type III-secretion effector genes (*exoT*, *exoY*) and antibiotic resistance

Antibiotic	exoT Resistant Gene + *n*(%)	exoT Susceptible Gene + *n*(%)	exoT Resistant Gene– *n*(%)	exoT Susceptible Gene– *n*(%)	exoT OR	exoT 95% CI	exoT *p*	exoT q	exoY Resistant Gene + *n*(%)	exoY Susceptible Gene + *n*(%)	exoY Resistant Gene– *n*(%)	exoY Susceptible Gene– *n*(%)	exoY OR	exoY 95% CI	exoY *p*	exoY q
Amikacin	16 (48%)	17 (52%)	13 (52%)	12 (48%)	0.869	0.31–2.46	1.000	1.000	16 (42%)	22 (58%)	13 (65%)	7 (35%)	0.392	0.13–1.20	0.167	0.373
Gentamicin	22 (67%)	11 (33%)	17 (68%)	8 (32%)	0.941	0.31–2.85	1.000	1.000	22 (58%)	16 (42%)	17 (85%)	3 (15%)	0.243	0.06–0.97	0.044	0.351
Tobramycin	17 (52%)	16 (48%)	11 (44%)	14 (56%)	1.352	0.48–3.84	0.606	0.969	15 (39%)	23 (61%)	13 (65%)	7 (35%)	0.351	0.11–1.08	0.097	0.373
Ceftazidime	28 (85%)	5 (15%)	19 (76%)	6 (24%)	1.768	0.47–6.63	0.504	0.969	29 (76%)	9 (24%)	18 (90%)	2 (10%)	0.358	0.07–1.85	0.299	0.478
Cefepime	30 (91%)	3 (9%)	18 (72%)	7 (28%)	3.889	0.89–16.97	0.083	0.221	30 (79%)	8 (21%)	18 (90%)	2 (10%)	0.417	0.08–2.18	0.468	0.614
Ciprofloxacin	27 (82%)	6 (18%)	15 (60%)	10 (40%)	3.000	0.91–9.89	0.081	0.221	26 (68%)	12 (32%)	16 (80%)	4 (20%)	0.542	0.15–1.97	0.538	0.614
Levofloxacin	27 (82%)	6 (18%)	19 (76%)	6 (24%)	1.421	0.40–5.08	0.745	0.994	28 (74%)	10 (26%)	18 (90%)	2 (10%)	0.311	0.06–1.59	0.187	0.373
Piperacillin/tazobactam	33 (100%)	0 (0%)	20 (80%)	5 (20%)	17.976	0.94–342.35	0.012	0.093	34 (89%)	4 (11%)	19 (95%)	1 (5%)	0.447	0.05–4.30	0.650	0.650

For each antibiotic, resistant and susceptible isolate counts plus proportions (%) are provided for gene-positive and gene-negative subgroups (*exoT* or *exoY*). Odds ratios (OR) and 95% CIs use the Woolf log method; zero cells were handled with a Haldane–Anscombe 0.5 continuity correction. Two-tailed Fisher’s exact test *p*-values were adjusted for eight comparisons per gene using Benjamini–Hochberg FDR (α = 0.05); *q*-values are reported. After adjustment, no *exoT* or *exoY* association reached significance (lowest *q* corresponds to *exoT* vs. piperacillin/tazobactam)

### PFGE results

**Fig. 2:**
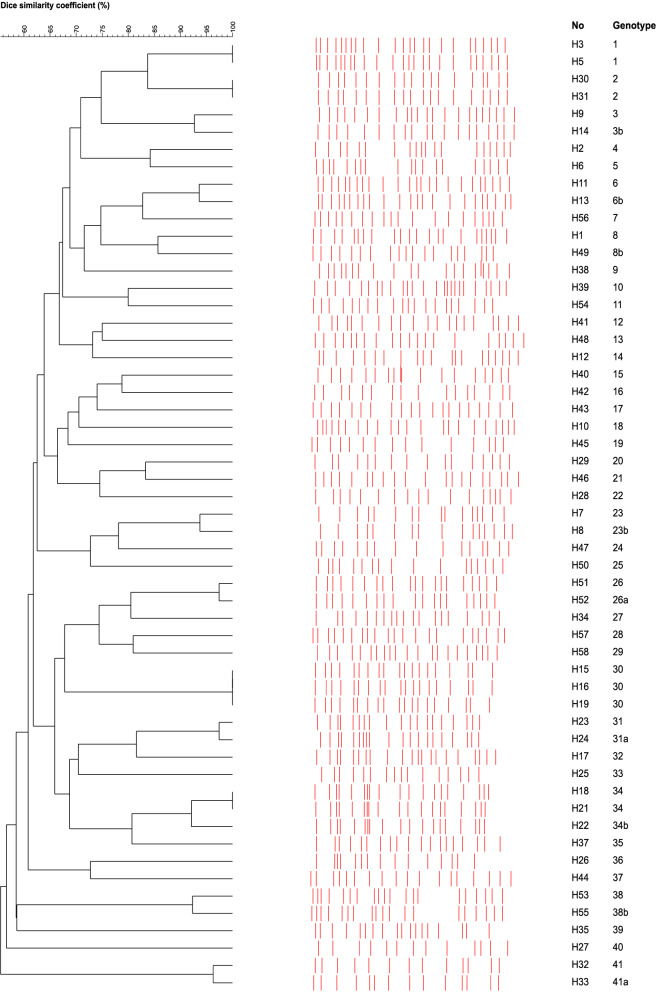
Schematic UPGMA dendrogram illustrating the PFGE-based genetic relatedness of carbapenem-resistant Pseudomonas aeruginosa isolates. Clustering was performed using the Dice similarity coefficient with a position tolerance of 1.5% and an optimization value of 1.0%. Isolates showing ≥85% similarity were considered closely related and assigned to the same cluster.

PFGE patterns were interpretable for 55/58 CRPA isolates; three isolates (H4, H20, H36) lacked evaluable banding and were excluded. No dominant outbreak PFGE type was detected. Using Dice similarity (position tolerance 2.0%, optimization 1.0%) and UPGMA (Unweighted Pair Group Method with Arithmetic Mean) clustering with an 85% similarity cut-off, 12 clusters comprising 26/55 (47%) isolates were identified (Fig. 2). The largest clusters (PFGE types 30 and 34) each contained three isolates; the remaining clusters (PFGE types 1, 2, 3, 6, 8, 23, 26, 31, 38, and 41) comprised two isolates each. Cluster membership did not segregate by sample origin; identical or highly similar PFGE types occurred in both respiratory and non-respiratory isolates. For example, PFGE type 31 (H23, H24; Surgical ICU) and PFGE type 38 (H53, H55; temporary COVID-19 wards) grouped phenotypically similar isolates but lacked definitive epidemiological linkage. PFGE type 34 (three isolates; Internal-medicine ICU) carried fewer virulence markers and showed broader antibiotic susceptibility, consistent with a less concerning local clone. Overall, the dendrogram supports a polyclonal CRPA population with occasional small clusters rather than a single epidemic lineage. Isolate-level PFGE types and associated metadata are detailed in Table 1.

## Discussion

Carbapenem-resistant *Pseudomonas aeruginosa* (CRPA) represents a critical global health challenge. In May 2024, the World Health Organization elevated CRPA from the ‘critical’ to the ‘high priority’ category on its ‘List of Bacterial Priority Pathogens’, underscoring the urgent need for investment in research, infection control, and treatment due to persistently high resistance rates in certain regions [31].

Respiratory tract isolates are frequently encountered in intensive care units. A study conducted in 2025 identified CRPA isolation as an independent predictor of respiratory failure, the requirement for mechanical ventilation, and extended hospital stays. Furthermore, the production of metallo-β-lactamase (MBL) in respiratory isolates significantly complicates treatment and infection control for these patients [32]. We attribute the high resistance rates observed in our study primarily to the fact that 50% of the isolates originated from Intensive Care Units (ICUs), where carbapenem usage is extensive. Additionally, high hospital admission rates may have led to increased antibiotic consumption, further driving resistance. The relatively low resistance to aminoglycosides, particularly amikacin, aligns with previous findings [33–35]. This is likely due to the specific substrate profile of aminoglycoside-modifying enzymes and the less frequent clinical use of this class compared to beta-lactams.

A significant clinical challenge is presented by the finding that 34% of isolates exhibited a pan-drug-resistant (PDR) profile. Treating these infections often necessitates last-resort agents such as colistin or novel combinations (e.g., ceftazidime-avibactam or cefiderocol), which were not included in our routine susceptibility panel. This high PDR rate highlights the urgent need to expand susceptibility testing panels in our hospital and emphasizes the critical necessity for stringent antibiotic stewardship to preserve remaining therapeutic options.

Carbapenemases were first identified in Europe in the 1990 s and have since proliferated at an alarming rate. These enzymes are predominantly plasmid-mediated, with KPC, VIM, IPM, NDM, and OXA-48 being the most common types. Carbapenemase production has been primarily detected in *Enterobacterales*, *Pseudomonas aeruginosa*, and *Acinetobacter baumannii* [36]. Researchers often prioritize phenotypic antibiotic resistance profiling prior to genotypic screening [6]. This stepwise approach is cost-effective, conserves specialized laboratory resources, and focuses molecular testing on isolates that are most likely to harbor specific resistance elements, optimizing workflow in resource-sensitive settings.

This study evaluated 58 carbapenem-resistant *P. aeruginosa* isolates and detected the *bla*VIM gene in only three (5%). No other carbapenemase genes were detected, nor was any significant gene association found. The low prevalence of carbapenemase genes suggests that the resistance profile of *Pseudomonas* in our setting is heterogeneous, likely driven by alternative mechanisms such as porin loss or efflux pump overexpression [5, 37]. These findings are consistent with a previous study reporting similarly low carbapenemase gene rates [38]. The exclusive detection of *bla*VIM aligns with literature highlighting the prevalence of *bla*VIM and *bla*IMP in *Pseudomonas* [5, 6]. While some studies report higher detection rates, our results parallel other studies conducted in our country. It has been predicted that genetic diversity could be revealed more comprehensively with a larger sample size [39]. Although all three *bla*VIM-positive isolates were obtained from respiratory tract samples, the small number of positive cases precludes a definitive statistical association.

In this study, 28 (48%) of the isolates were *mexY*-positive. Similar rates of *mexY* positivity have been reported elsewhere [40]. Only one isolate exhibited concurrent *bla*VIM and *mexY* positivity. Although this isolate was from a respiratory sample, no statistically significant relationship was found between *mexY* positivity and specimen source (Table 3).

Virulence factors such as Exotoxin A (*exoA*), T3SS effectors (*exoS*,* exoT*,* exoY*,* exoU*), and biofilm formation are central to *P. aeruginosa* pathogenicity. These factors are regulated by the Quorum Sensing (QS) system, specifically the *las* and *rhl* genes [13]. In our cohort, QS genes were highly prevalent (*rhlR* 95%; *lasR* 91%), and virulence genes *exoY*, *exoT*, and *algD* were also frequently detected. This indicates a high pathogenic potential and the capacity for coordinated attacks via environmental signal detection. The Las system is effective in the early stages of biofilm formation, whereas the Rhl system functions in later stages [13]. Some studies suggest that *lasR*-deficient strains are less virulent and that *lasR* mutations correlate with decreased biofilm formation [41]. The high detection rate of QS and virulence genes, combined with the robust resistance profile in our study, may be linked to the high proportion of ICU isolates.

Literature indicates that *exoT* and *exoY*, two genes belonging to the T3SS family, are frequently detected at rates above 90% [12]. Consistent with other studies [12, 42], we found *exoA* at a lower frequency than *exoT* and *exoY*. While *algD* has been detected at rates up to 98% in some reports, our detection rate of approximately 58% aligns with others. Importantly, in this cohort, the T3SS effector genes *exoT* and *exoY* did not co-segregate with resistance to β-lactams, aminoglycosides, or fluoroquinolones. Mechanisms mediating drug escape (efflux, porin loss, carbapenemase) appear to evolve independently of these effectors. While some studies report higher virulence factor prevalence in respiratory samples [12], we found no statistically significant relationship between virulence gene prevalence and specimen type (Table 3). Furthermore, after False Discovery Rate (FDR) adjustment, no *exoT* or *exoY* association with antibiotic resistance remained significant (lowest q = 0.093 for *exoT*–TZP) (Table 4). In contrast, Tang et al. [43] reported significant differences in resistance across virulence groups, and Ullah et al. [44] noted lower imipenem/TZP resistance in *exoY*-positive isolates; however, these studies may reflect clone-specific contexts or uncorrected statistical noise. Together, Tables 3 and 4 demonstrate a sample-type-independent, polyclonal gene pool and a virulence-gene-independent resistance profile in our CRPA isolates.

These findings support Horizontal Gene Transfer (HGT), rather than clonal expansion, as the primary driver of resistance dissemination. Many clustered isolates originated from ICUs, yet clusters cut across different ICUs and outpatient units, suggesting sporadic introductions rather than sustained ward-specific outbreaks. Pan-drug-resistant profiles appeared on multiple, genetically distant branches, further supporting the role of HGT (e.g., integrons, plasmids) over clonal spread. Similar epidemiological results were reported in a multi-center study in Poland [45], although this contrasts with reports from Kosovo [46] and other European regions [47] where clonal clusters were more frequent in ICUs. A study conducted in our country [48] observed similar genomic diversity without a correlation between genotypic and phenotypic characteristics. Another study reported a clustering rate of 53%, paralleling our findings. The relatively high recombination frequency of *P. aeruginosa* likely contributes to this multi-clonal population structure [49].

Infection control strategies should therefore focus on broad barriers to horizontal gene transfer—such as hand hygiene, environmental decontamination, and antibiotic stewardship—rather than targeting a single clone. Surveillance schemes would benefit from Whole Genome Sequencing (WGS) to disentangle the relative contributions of plasmid-borne resistance versus clonal spread. Our integrative re-analysis, backed by stringent BH-FDR control, aligns with recent genomic epidemiology reports [50] portraying CRPA as a recombinant, “gene-flux” community.

Our study has several limitations. First, as a single-center study with a relatively small sample size (*n* = 58), the generalizability of the findings may be limited. Second, plasmid extraction and replicon typing were not performed; thus, the role of specific plasmids in HGT was inferred from PCR and PFGE patterns rather than direct analysis. Third, the retrospective design prevented a precise temporal analysis of transmission chains due to the unavailability of detailed patient movement data. Finally, while PFGE provides robust typing, future surveillance efforts should incorporate WGS to provide higher-resolution data on specific resistance mutations and clonal dynamics.

In conclusion, this study underscores the need to blend high-resolution genomics with disciplined statistics to accurately disentangle true resistance-virulence synergies from epidemiological coincidences.

## Supplementary Information

Supplementary Material 1.

## Data Availability

All data supporting the results are provided within the article and its Additional files. Machine-readable raw counts (table3_counts.csv, table4_counts_corrected.csv) and the analysis script (reproduce_analysis_corrected.py) with the environment file (environment.yml) are packaged in Additional file 4 (ZIP) and archived on Zenodo (https://doi.org/10.5281/zenodo.16029015), enabling full reproducibility. De-identified isolate-level metadata underlying Table 1 are available from the corresponding author upon reasonable request; no patient-identifying information is included.

## Abbreviations

AK: Amikacin
BH: Benjamini–Hochberg false discovery rate correction
CAZ: Ceftazidime
CIP: Ciprofloxacin
CI: Confidence interval
CN: Gentamicin
CRPA: Carbapenem-resistant Pseudomonas aeruginosa
EUCAST: European Committee on Antimicrobial Susceptibility Testing
FDR: False discovery rate
FEP: Cefepime
HGT: Horizontal gene transfer
ICU: Intensive care unit
I: Susceptible, increased exposure (EUCAST category)
KPC: Klebsiella pneumoniae carbapenemase
LEV: Levofloxacin
MDR: Multidrug-resistant
NDM: New Delhi metallo-β-lactamase
OR: Odds ratio
OXA-48: OXA-48-like class D carbapenemase
PCR: Polymerase chain reaction
PFGE: Pulsed-field gel electrophoresis
PDR: Pan-drug-resistant
q-value: FDR-adjusted p-value
R: Resistant (EUCAST category)
SIM: Seoul imipenemase
S: Susceptible (EUCAST category)
SpeI: Type II restriction endonuclease SpeI
T3SS: Type III secretion system
TOB: Tobramycin
TZP: Piperacillin/tazobactam
VIM: Verona integron-encoded metallo-β-lactamase
ENT: Ear, nose and throat
TA: Tracheal aspirate
