# Supplementary material for: Genotypic and phenotypic landscape of carbapenem-resistant Pseudomonas aeruginosa isolated from respiratory and non-respiratory samples in a tertiary hospital
Source: BMC Microbiol. 2026 Mar 14;26:583. doi: 10.1186/s12866-026-04756-8 (PMC13325778; doi:10.1186/s12866-026-04756-8)
Supplement: Supplementary file 1 — Supplementary Material 1. [file 12866_2026_4756_MOESM1_ESM.zip › Additional file 3.docx]

**Additional Files**

**Genotypic and Phenotypic Landscape of Carbapenem-Resistant Pseudomonas aeruginosa Isolated From Respiratory and Non-Respiratory Samples in a Tertiary Hospital**

**Overview and file list (for publication as provided):**

- Supplementary Table S1. PCR cycling conditions.
- Supplementary Table S2: Raw counts used for the exoT/exoY–antibiotic analyses (mirrors table4_counts_corrected.csv).
- Supplementary Figure S1 (PNG/PDF): BH-adjusted q-values for gene-prevalence comparisons between respiratory and non-respiratory isolates (two-tailed Fisher’s exact tests; Benjamini–Hochberg FDR 0.05).
- Supplementary Figure S2 (PNG/PDF): BH-adjusted q-values for exoT/exoY–antibiotic associations across eight agents (Fisher’s exact tests; FDR 0.05).
- Supplementary File S3 (ZIP): reproduce_analysis_corrected.py, table3_counts.csv, table4_counts_corrected.csv, environment.yml (full reproducibility).
- Note: Manuscript Tables 1–4 are part of the main article and are not reproduced here.

**Supplementary Table S1.** PCR cycling conditions.

| **Reaction** | **First denaturation** | **Denaturation** | **Annealing** | **Extension** | **Final extension** | **Cycles** | **Reference** |
| --- | --- | --- | --- | --- | --- | --- | --- |
| **IMP** | 94°C (5 min) | 94°C (1 min) | 54°C (1 min) | 72°C (50 s) | 72°C (7 min) | 34 | [16] |
| **VIM** | 94°C (5 min) | 94°C (30 s) | 57°C (30 s) | 72°C (45 s) | 72°C (7 min) | 32 | [16] |
| **OXA-48** | 94°C (5 min) | 94°C (1 min) | 55°C (1 min) | 72°C (50 s) | 72°C (7 min) | 34 | [17] |
| **KPC** | 94°C (5 min) | 94°C (1 min) | 55°C (1 min) | 72°C (1 min) | 72°C (7 min) | 34 | [18] |
| **NDM** | 94°C (3 min) | 94°C (45 s) | 52°C (1 min) | 72°C (1.5 min) | 72°C (7 min) | 34 | [19] |
| **SIM** | 94°C (10 min) | 94°C (30 s) | 52°C (40 s) | 72°C (50 s) | 72°C (5 min) | 36 | [20] |
| **mexY** | 94°C (30 s) | 94°C (5 s) | 58°C (10 s) | 72°C (20 s) | 72°C (5 min) | 45 | [21] |
| **exoA** | 94°C (2 min) | 94°C (1 min) | 68°C (1 min) | 72°C (1 min) | 72°C (7 min) | 30 | [21] |
| **exoT** | 94°C (2 min) | 94°C (30 s) | 58°C (30 s) | 68°C (1 min) | 68°C (7 min) | 36 | [21] |
| **exoY** | 94°C (2 min) | 94°C (30 s) | 58°C (30 s) | 68°C (1 min) | 68°C (7 min) | 36 | [22] |
| **algD** | 94°C (5 min) | 94°C (45 s) | 53°C (1 min) | 72°C (1 min) | 72°C (7 min) | 30 | [23] |
| **lasR** | 94°C (2 min) | 94°C (40 s) | 60°C (1 min) | 72°C (2 min) | 72°C (10 min) | 30 | [23] |
| **rhlR** | 94°C (2 min) | 94°C (40 s) | 60°C (1 min) | 72°C (2 min) | 72°C (7 min) | 30 | [24] |

**Supplementary Table S2.** Raw counts for exoT/exoY–antibiotic associations (as used in Table 4 analyses).

| **Antibiotic** | **S_exoT_pos** | **R_exoT_pos** | **S_exoT_neg** | **R_exoT_neg** | **S_exoY_pos** | **R_exoY_pos** | **S_exoY_neg** | **R_exoY_neg** |
| --- | --- | --- | --- | --- | --- | --- | --- | --- |
| **Amikacin** | 17 | 16 | 12 | 13 | 22 | 16 | 7 | 13 |
| **Gentamicin** | 11 | 22 | 8 | 17 | 16 | 22 | 3 | 17 |
| **Tobramycin** | 16 | 17 | 14 | 11 | 23 | 15 | 7 | 13 |
| **Ceftazidime** | 5 | 28 | 6 | 19 | 9 | 29 | 2 | 18 |
| **Cefepime** | 3 | 30 | 7 | 18 | 8 | 30 | 2 | 18 |
| **Ciprofloxacin** | 6 | 27 | 10 | 15 | 12 | 26 | 4 | 16 |
| **Levofloxacin** | 6 | 27 | 6 | 19 | 10 | 28 | 2 | 18 |
| **Piperacillin/tazobactam** | 0 | 33 | 5 | 20 | 4 | 34 | 1 | 19 |

Note: The authoritative machine-readable counts are provided in table4_counts_corrected.csv within Additional file 3 (ZIP).

**Figure captions**

**Supplementary Figure S1.** BH-adjusted q-values for gene prevalence by specimen source (respiratory vs non-respiratory). Bars display Benjamini–Hochberg–adjusted q-values for 11 genes; the dashed line marks the FDR threshold (q = 0.05). No comparison remained significant after adjustment.

**Supplementary Figure S2.** BH-adjusted q-values for exoT/exoY–antibiotic associations. Bars show BH-adjusted q-values for each gene–antibiotic pair; the dashed line marks the FDR threshold (q = 0.05). No association survived FDR correction (lowest q = 0.093 for TZP in the exoT analysis).

**Reproducibility notes**

All analyses can be reproduced with Python 3.11 using the files provided with this Additional Files. Zero cells in 2×2 tables are handled by the Haldane–Anscombe continuity correction; 95% CIs for odds ratios use the Woolf log method. Unless otherwise stated, S = susceptible + intermediate (EUCAST v11.0, 2021).

Example usage:

conda env create -f environment.yml

conda activate crpa_analysis

python reproduce_analysis_corrected.py

**Conda environment (environment.yml)**

name: crpa_analysis
channels:
 - defaults
dependencies:
 - python=3.11
 - pandas
 - numpy
 - matplotlib
 - scipy
 - statsmodels
 - python-docx
