# Supplementary figures and images for: Genotypic and phenotypic landscape of carbapenem-resistant Pseudomonas aeruginosa isolated from respiratory and non-respiratory samples in a tertiary hospital
Source: BMC Microbiol. 2026 Mar 14;26:583. doi: 10.1186/s12866-026-04756-8 (PMC13325778; doi:10.1186/s12866-026-04756-8)

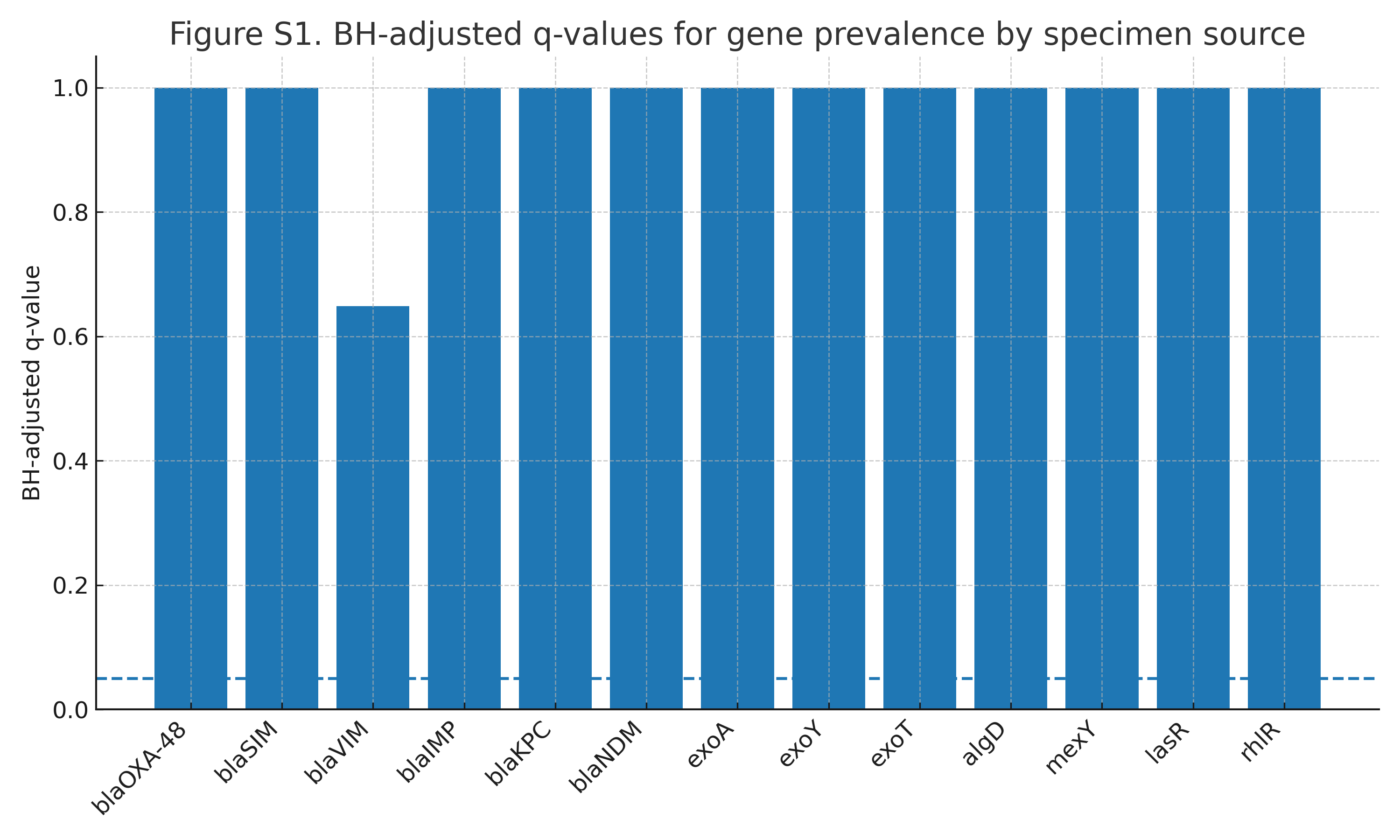

Supplement: Supplementary file 1 — Supplementary Material 1. [file 12866_2026_4756_MOESM1_ESM.zip › Additional file 1.png]

Figure S1. BH-adjusted q-values for gene prevalence by specimen source

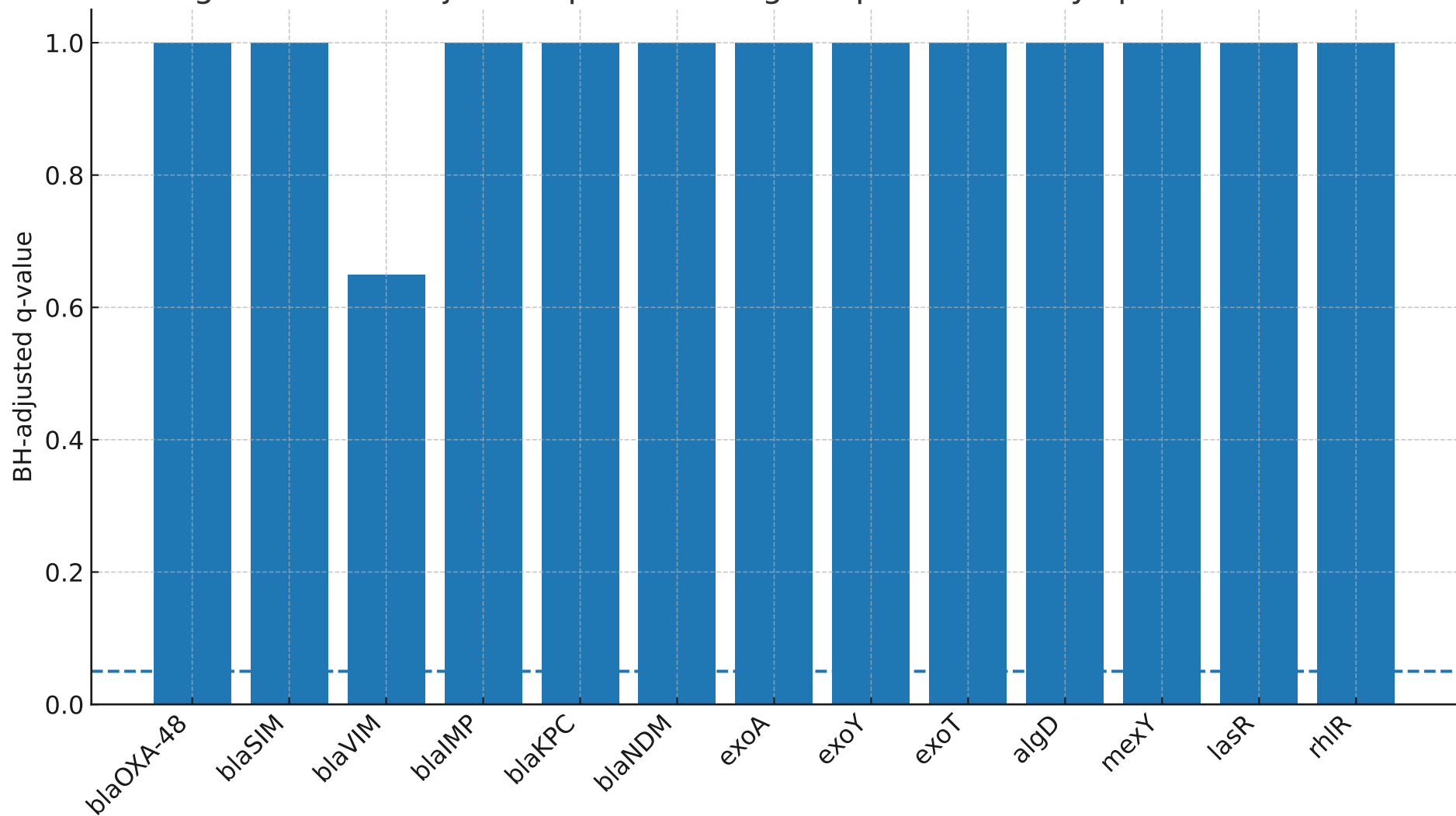

Supplement: Supplementary file 1 — Supplementary Material 1. [file 12866_2026_4756_MOESM1_ESM.zip › Additional file 1.pdf]

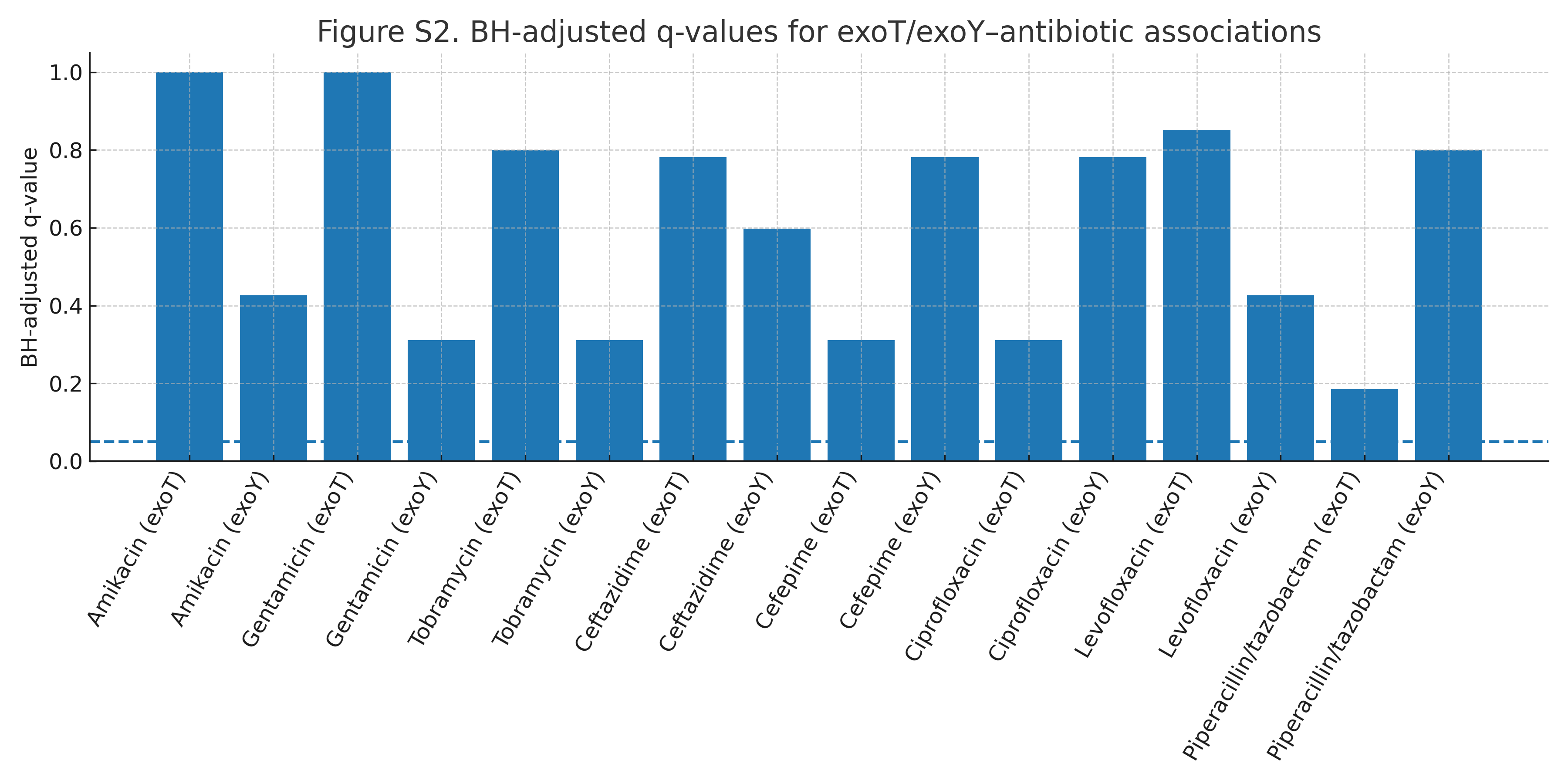

Supplement: Supplementary file 1 — Supplementary Material 1. [file 12866_2026_4756_MOESM1_ESM.zip › Additional file 2.png]

Figure S2. BH-adjusted q-values for *exoT*/*exoY*-antibiotic associations

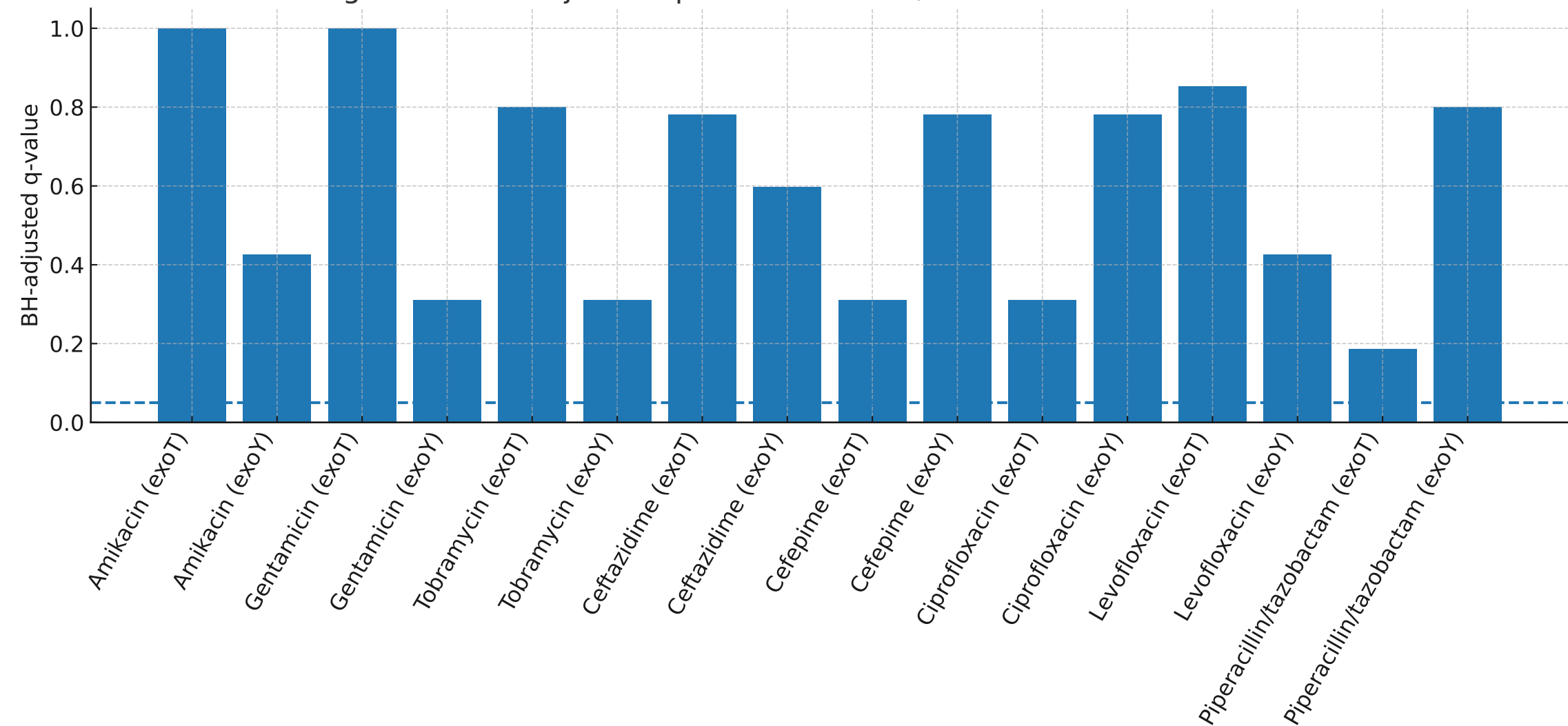

Supplement: Supplementary file 1 — Supplementary Material 1. [file 12866_2026_4756_MOESM1_ESM.zip › Additional file 2.pdf]
